# Characterization and energy recovery of fuels from medical waste via thermal pyrolysis

**DOI:** 10.1016/j.heliyon.2025.e42599

**Published:** 2025-02-12

**Authors:** Adnan Abedeen, Md. Shameem Hossain, A. N. M. Mizanur Rahman

**Affiliations:** a Institute of Environment and Power Technology, Khulna University of Engineering & Technology, Khulna, 9203, Bangladesh; b Department of Energy Science and Engineering, Khulna University of Engineering & Technology, Khulna, 9203, Bangladesh; c Department of Mechanical Engineering, Khulna University of Engineering & Technology, Khulna, 9203, Bangladesh

**Keywords:** Pyrolysis, Medical waste, GC-MS, FT-IR, TGA-DTG curve, Pyrolytic oil, Alternative fuel

## Abstract

This research studies the potentiality of recovering alternative fuel from the pyrolysis of syringe waste (SW) and saline bottle waste (SBW). Plastic-based medical wastes can cause severe environmental and human health damage if not properly managed. Lab-scale experiments were conducted in a batch-type fixed-bed reactor by fluctuating the temperature within the 0–600°C range at an interval of 50°C. The effect of temperature on product yield has been investigated. Various properties of pyrolytic oil extracted from SW and SBW, such as density, kinematic viscosity, pour point, boiling point, and cloud point were measured. The respective values were in the range of 726–758 kg/m^3^, 3.19 to 4.75 cSt, −12 to −16°C, 86–95°C, −2 to −5°C and GCV was around 42–44 MJ/kg. The GCV of pyrolytic char was around 42–43 MJ/kg. GC-MS and FT-IR tests suggested the presence of higher amounts of alcohols and organosilicons in pyrolytic oils, which evolved from SW and SBW, respectively. TGA-DTG curve indicated the thermal fracture range of SW and SBW pyrolytic oil was 50–280°C. Oil and char obtained in this study can be used as alternative fuel or chemical feedstock in different industries after some treatment. Results also showed that the properties are like low-grade liquid fuels and high-grade solid fuels. During COVID-19 and post-pandemic, a large amount of clinical waste has been used, and thus, it has become colossal waste. Pyrolysis of such waste (syringe/saline bottle) can reduce environmental contamination to a degree as well as be a substitute source of energy.

## Highlights

• Collected plastic medical waste were pyrolyzed in a batch type fixed bed reactor.
• Maximum production of pyrolytic oil and char were found at temperatures of 250°C and 150°C respectively.
• GCV of pyrolytic oil and char from waste syringes/saline bottles were around 42-44 MJ/kg and 42-43 MJ/kg respectively.
• Presence of useful chemical compounds were found in pyrolytic oil through GC-MS analysis which could be used in various industries.
• Pyrolytic char could be used as activated carbon.

## Graphical abstract

**Figure:**
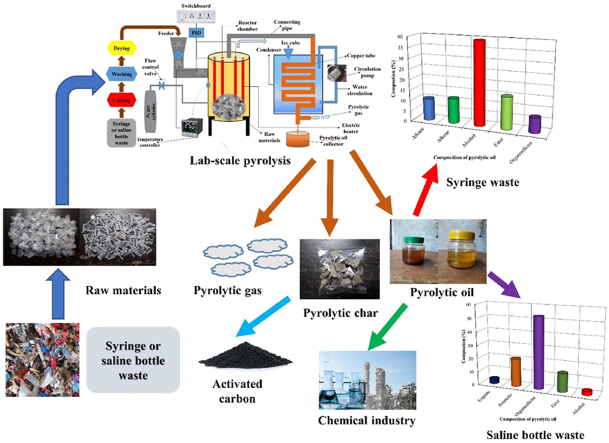

## 1 Introduction

**Figure 1:**
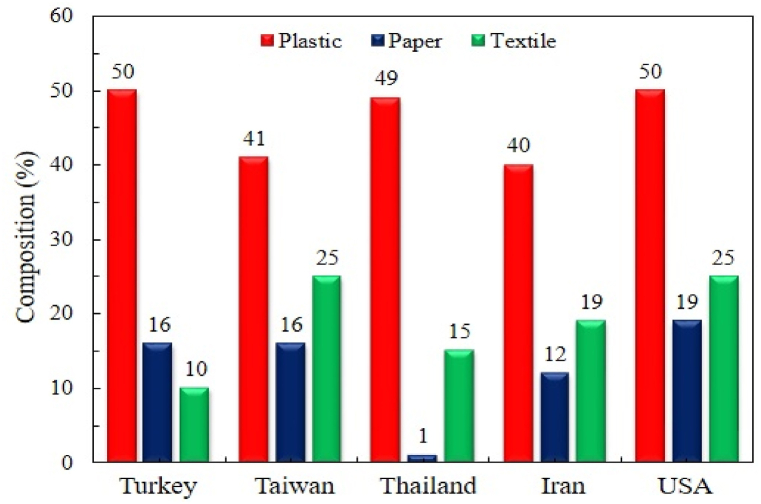
Medical waste composition of different countries

The fossil fuel crisis has led mankind to focus on developing new and alternate energy sources. Proper waste management and strategic policy are also essential to sustainable development [1]. Management of medical waste is considered an essential matter worldwide [2]. Healthcare facilities are increasing rapidly, which sequentially increases the quantity of medical waste production in developing countries. If not carefully managed, medical waste threatens persons, communities, and the surroundings. Globally, around 75–90% of healthcare wastes are non-toxic, originating from healthcare organizations' maintenance and managerial functions [3]. The composition and generation of healthcare waste for some selected countries [4,5] are shown in Fig. 1 and Table 1, respectively.

**Table 1:** Healthcare waste generation rates in selected countries

Country	HCWGR (kg/Bed/Day)	References
Bangladesh	1.24	[11,12]
India	1.55	[13]
Pakistan	2.07	[14]
Nepal	0.5	[15]
Malaysia	1.9	[15]
Indonesia	0.75	[16]
Thailand	2.05	[17]
Iran	3.04	[15,18]
France	3.3	[14]
United Kingdom	3.3	[14]
Germany	3.6	[19]
China	4.03	[20,21]
Canada	8.2	[22]
United States of America	8.4	[14,22,23]

Many factors are accountable for the variability of the healthcare waste generation rate (HCWGR) [6]. Healthcare waste (HCW) generation is sharply escalating in developing countries because of improved access to medical services [7]. Landfilling is not an appropriate option for discarding plastic medical wastes because of their prolonged degradation rates. Mechanical recycling can be an effective process with some limitations [8]. Pyrolysis is one of the most efficient and promising techniques for liquid fuel from plastic-based waste [9]. It produces valuable hydrocarbons, which are used as fuel and different types of feed materials for various chemical industries [10].

During the COVID-19 pandemic worldwide, there was a considerable escalation in the consumption of medical waste. Increased biomedical waste generation has created enormous difficulties for waste management approaches, promoting grave concerns regarding the possible hazards of inappropriate disposal. If not appropriately supervised, contagious biomedical waste could damage community health. Efficient methods for safely and effectively handling medical waste are essential to alleviate these risks and protect human well-being [[24], [25], [26], [27], [28], [29], [30]].

A case study on Khulna City Corporation (KCC) shows an average healthcare waste production of about 1.29 kg/bed/day [31]. A similar study on Jashore municipality shows an average healthcare waste generation rate of 1.59 kg/bed/day [32]. Another study on Rajshahi City Corporation (RCC) shows an average healthcare waste production of 1.54 kg/bed/day [33]. These case studies found that the average production of healthcare waste in different big cities of Bangladesh is approximately 1.5 kg/bed/day.

Limited available scholarly research demonstrated the proficient production of alternative fuel from the pyrolysis of medical wastes. Fahim et al. examined the synergistic interactions, pyrolysis reaction mechanisms, and pyrolytic gas release behavior of regular components of syringe waste, cotton swab sticks, medical gloves, medical waste, and their blends by applying cutting-edge methods, including TG-MS, TG-FT-IR, TG, and various kinetic models [34]. Weijie et al. studied the characterization and verification of pyrolysis reaction mechanisms of mask faces, medical mask belts, and infusion tubes through TGA, vibrational spectroscopy, TG-FT-IR, and py-GCMS techniques [35]. Ziyi et al. aimed to categorize pyrolysis behaviors, drivers, reaction mechanisms, products, and pathways of syringe waste and medical bottles using Py-GC/MS and TG-FT-IR considering their degradation stage, conversion degree and different heating rates [36]. Vasile et al. performed pyrolysis of disposable syringes and characterized the reaction products by density, gas-chromatography, aniline point analysis, refractive indices, and spectroscopic methods [37]. Deng et al. studied thermogravimetric analysis and various kinetic parameters on pyrolysis of some typical medical waste compositions [38]. Zhu et al. analyzed the pyrolysis of some known medical wastes using a thermogravimetric analyzer combined with Fourier transform infrared spectroscopy (TG-FT-IR) [39]. Bernardo et al. examined the physicochemical characteristics of pyrolytic chars produced from the co-pyrolysis of PP, PE, and PS plastic wastes, pine biomass, and scrap tires [40]. Dash et al. studied the thermolysis of syringe waste and the prospect of hydrocarbon production [41]. Ahmad et al. compared the characteristics of the oil produced from the pyrolysis of Polypropylene (PP) and High-density polyethylene (HDPE) with Diesel and Gasoline [42]. Pramanik et al. studied the production of pyrolytic oil from waste polyethylene using a specially designed semi-batch reactor. The pyrolytic oil produced was investigated and characterized for valuable properties such as API gravity, carbon residue, fire point, heating value, flash point, and proximate analysis [43]. Som et al. investigated the potentiality of beneficial product recovery from plastic medical waste (PMW) through pyrolysis. Polypropylene (PP) and high-density polyethylene are the main components of syringe and saline bottle waste [8].

Pyrolysis of various wastes is commonly conducted for energy recovery as the products from pyrolysis usually have high-quality fuel properties. Some valuable properties of pyrolytic oil and char make them favorable as raw materials for several industrial sectors. The heating value of the pyrolytic gas generated from slow pyrolysis is about 10–15 MJ/m^3^ [44]. The heating value of the pyrolytic gas derived from Polypropylene (PP) and Polyethylene (PE) varied from 42 to 50 MJ/kg [45]. The quality of pyrolytic oil as a fuel can be identified by measuring its physical properties, such as density, viscosity, boiling point, pour point, cloud point, and flash point [46]. The average heating value of pyrolytic oils generated from mixed plastic waste is around 40 MJ/kg [47]. A significant amount of toluene, styrene, and ethylbenzene is present in waste plastic pyrolytic oil and can be used as chemical feedstock in various chemical industries [48]. Pyrolytic oil from polyethylene terephthalate (PET) is less efficient because of its highly acidic nature [49]. Pyrolytic char produced from the pyrolysis of waste materials has a heating value of about 34 MJ/kg [50], which is comparable to bituminous coal.

## 2 Materials and methods

### 2.1 Raw materials

**Figure 2:**
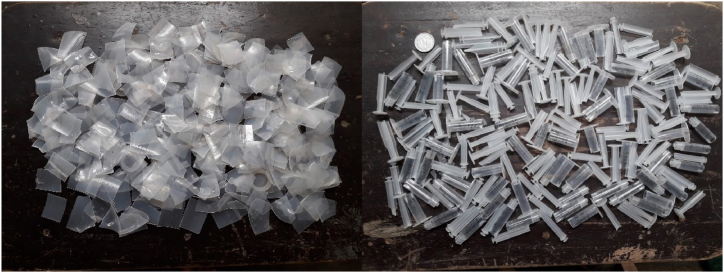
Chopped SW and SBW

Various equipment, chemicals, and materials were used to transform SW/SBW into pyrolytic gas, oil, and char. The raw materials used in this study for pyrolysis were disposable syringes and saline bottles collected from the local clinics and hospitals of Khulna city, Bangladesh. The wastes were then washed thoroughly with water and detergent to get rid of blood, mud, and other contaminants adhered to them. The washed raw materials were sun-dried, and then the sun-dried raw materials were chopped with a scissor into small pieces, as shown in Fig. 2.

### 2.2 Characterization of raw materials

Proximate and ultimate analysis of the raw feedstock have been identified by using a muffle furnace (SX-7-10D, USA) at 950°C and a CHNS elemental analyzer, varioMicro V1.6.1, GmbH, Germany, respectively. TGA analysis was done using a TGA-50H detector in Shimadzu, Japan. GC-MS investigations have been experimented with by GCMS-TQ8040. FT-IR tests have been conducted by IRTracer-100, Shimadzu, Japan. The gross calorific value (GCV) of the raw feedstock was determined by an Oxygen Bomb Calorimeter, Infitek, USA.

### 2.3 Experimental setup and procedure

**Figure 3:**
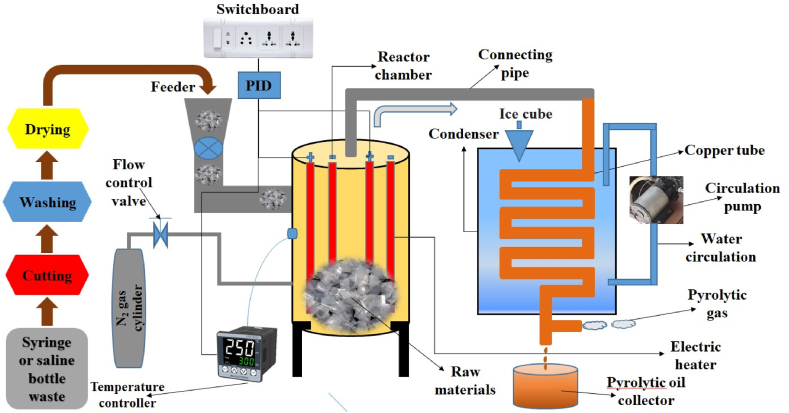
Schematic diagram of the experimental set-up

The main reactor chamber was cylindrical in shape and constructed of stainless steel, which had a length of 27.0 cm. The external diameter of the chamber was 22.7 cm, and the internal diameter was 22.0 cm. Fig. 3 shows the schematic diagram of the experimental setup. One end of the reactor chamber was closed, and the other was used as the feeding end. The reactor chamber was wrapped with asbestos rope for thermal insulation, so the heat could not come out quickly. The inside temperature of the reactor was recorded by K-type thermocouple sensors with a display facility. Firstly, 1 kg of shredded sample (SW or SBW) was measured using a weighing balance. The sample weighed was then charged into the reactor by opening the top cover and was heated by three U-shaped electric heaters arranged uniformly inside the reactor. The burning of raw materials took place in an oxygen-free atmosphere. Nitrogen gas was purged through the reactor chamber to ensure the complete non-existence of oxygen. The average heating rate maintained in this study was approximately 10°C/min. The combustion temperature range was 0–600°C for each SW and SBW experiment. The reaction time for each temperature was 30 minutes. The raw materials were decomposed and converted to gaseous substances as the temperature increased. The produced gas was passed through the connecting pipe to the condenser chamber. The icy water was circulated through a circulation pump to condense the gas circulating through copper tubes.

The non-condensable gases were flared into the open air. The heating was continued until all the gases came out. Generated pyrolytic oil came out beneath the condenser, was collected in a pot, and filtered further to separate and calculate wax and liquid oil. After completion of the process, the heater was turned off, and the reactor was cooled down. The remaining solid part was pyrolytic char mixed with a minimal amount of ash and was collected. First, the collected char was screened to eliminate the larger particles and collect the smaller, finer particles. To dismiss ash and other soluble contaminants, the collected char was washed with water several times.

To calculate the yields of pyrolytic products, after the completion of one operation, we opened the reactor and collected solid char. Then, we sum up the total weight of liquid oil products and solid char products. Finally, we have calculated the weight of gaseous products by deducting the weight of oil and char products from the initial feedstock. The weight of the gaseous products (W_g_) was calculated from the equation as given below:

W_g_ = W_f_ – (W_c_ + W_o_)

Where W_f_ is the weight of feedstock inserted into the reactor, W_c_ is the weight of solid char gained after one successful operation, and W_o_ is the weight of liquid oil gained after one successful operation [8].

### 2.4 Characterization methods of pyrolytic oil

The physical properties such as density, kinematic viscosity, pour point, boiling point, and cloud point of pyrolytic liquids from SW and SBW were determined based on ASTM D4052, D445, D97, D1120, and D5773, respectively. The results were compared with previous studies on gasoline and diesel fuel properties.

## 3 Results and discussion

### 3.1 Proximate and ultimate analysis of raw materials

When fuel samples are burnt under given conditions, a certain percentage of the fuel that burns in a gaseous state (volatile matter), in the solid state (fixed carbon), and the percentage of inorganic waste material that is almost inert (ash), the moisture content is the amount of water particle in the sample given as a percentage of the sample's original (wet) weight. It is, therefore, of fundamental importance for biomass fuel mainly. Thus, they are essential criteria for understanding fuel properties precisely. Table 2 illustrates the proximate and ultimate analysis of raw SW and SBW.

**Table 2:** Proximate and ultimate analysis of raw syringe and saline bottle waste.

Proximate Analysis (wt%)			Ultimate Analysis (wt%)		
Parameters	SW	SBW	Parameters	SW	SBW
Fixed Carbon (%)	34.06	36.13	Carbon (C)	84.66	67.50
Volatile Matter (%)	61.78	60.56	Hydrogen (H)	16.11	6.76
Moisture Content (%)	1.25	0.97	Nitrogen (N)	1.54	12.38
Ash Content (%)	2.91	2.34	Sulfur (S)	0.00	10.47

Table 2 shows that the fixed carbon percentage is higher in SBW than in SW, while the presence of volatile matter is slightly higher in SW. Moisture and ash content is quite similar in both raw materials. However, elemental carbon and hydrogen percentages are higher in SW than in SBW. At the same time, the nitrogen percentage is lower in SW. The sulfur percentage is higher in SBW, and elemental sulfur is absent in SW.

### 3.2 Thermogravimetric analysis (TGA) and TGA-DTG curve

**Figure 4:**
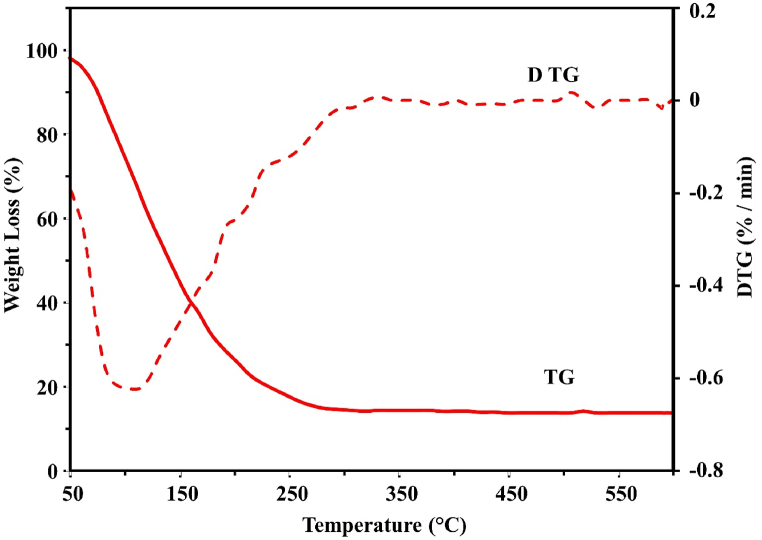
TGA-DTG curve of pyrolytic oil derived from SW

**Figure 5:**
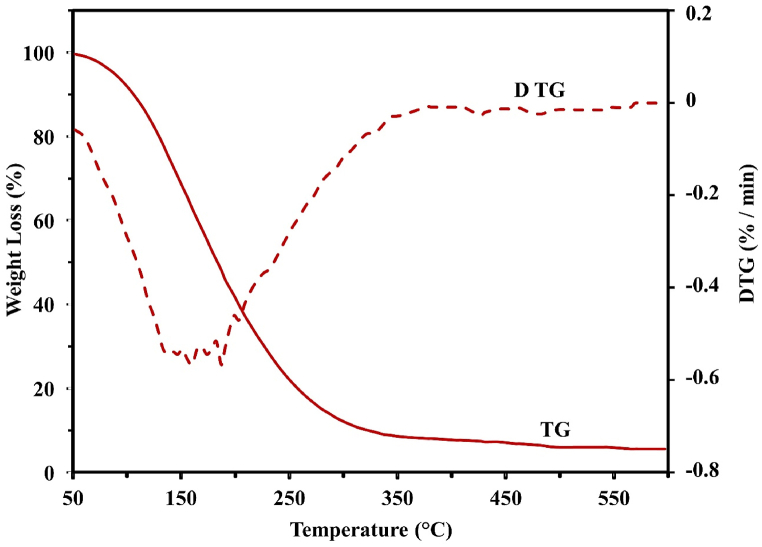
TGA-DTG curve of pyrolytic oil derived from SBW

The TGA data facilitates the evaluation of material's thermal stability and decomposition behavior under controlled conditions in inert or oxidizing environments. Pyrolytic oil extracted from SW and SBW were represented in the TGA graphs as they were heated at 10°C/min in an oxidizing environment. Figure 4, Figure 5 illustrate the weight loss versus thermogravimetric (TG) and DTG curves. Fig. 4 shows that, for SW, an initial rapid weight loss begins at around 50°C and continues until approximately 150°C. This stage usually corresponds to chemicals at a low boiling point and moisture evaporation. Between 150°C and 250°C, there is a progressive rise in weight loss, which is probably due to the breakdown of less volatile organic compounds. Over 250°C, the weight loss stabilizes and continues until 550°C, meaning that little to no residual char remains after nearly all the decomposing material has volatilized or broken down. Remarkably, there is a notable reduction in weight at 150°C, suggesting that most of the breakdown occurs at lower temperatures in the temperature range under investigation. The TGA analysis shows that the decomposition temperatures at these heating rates are lower, possibly due to various stabilizers, plasticizers, and additives in the plastic components [51].

The dashed red line on the DTG curve depicts the weight loss rate as a temperature function. A peak at approximately 150°C represents the temperature at which the maximum rate of breakdown takes place and correlates with the TG curve's rapid weight loss. Another peak indicates a subsequent decomposition phase at about 250°C. Once the temperature surpasses 250°C, the rate of weight loss becomes negligible and eventually stops, signifying the completion of the primary thermal breakdown processes. At a temperature increase of 10°C/min from 50°C to 150°C, Fig. 5 shows a rapid initial weight loss with moisture, low boiling point chemicals, and a continuous N_2_ flow. A more gradual weight loss between 150°C and 250°C indicates the breakdown of less volatile organic compounds. The temperature at which 50% weight loss occurs is approximately 150°C for SBW pyrolytic oil, with the decomposition process completing at 340°C and negligible residual char remaining. The DTG curve exhibits a notable peak around 150°C, which aligns with the maximum rate of disintegration noted in the TG curve. The rate of weight loss dramatically decreases after 250°C and approaches zero, signifying the conclusion of the main thermal decomposition processes. A secondary peak around 250°C indicates another phase of decomposition. Pyrolytic oil from syringe waste decomposes at a lower temperature than saline bottle waste, leaving little residue after complete thermal degradation. It is thermally fractured in the 50–280°C range under oxidizing conditions.

### 3.3 Effect of temperature on product yield

**Figure 6:**
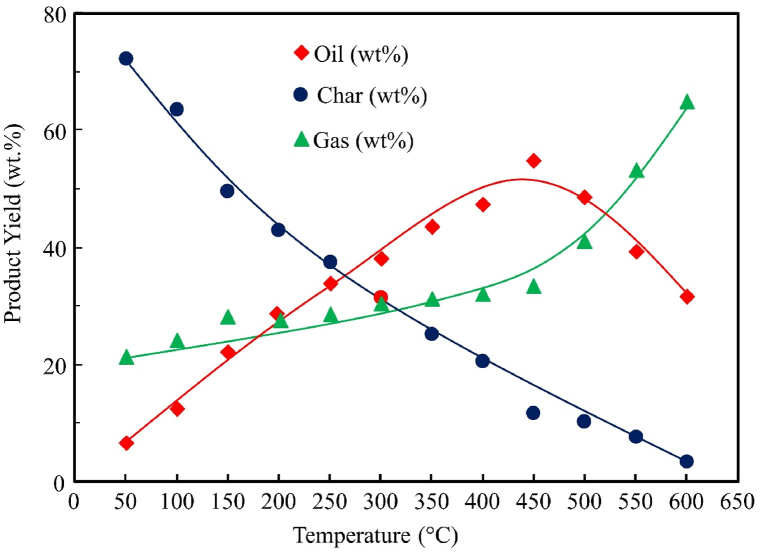
Temperature effect on product yield for SW

**Figure 7:**
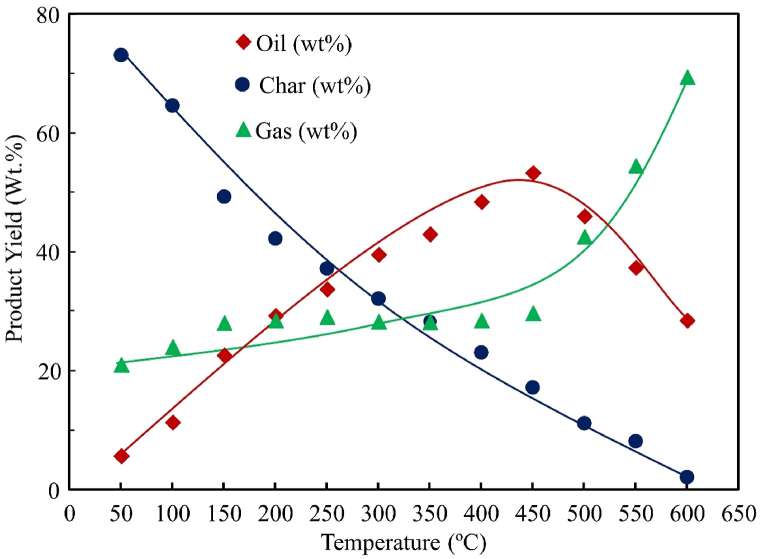
Temperature effect on product yield for SBW

Three sets of experiments were performed in this study, each one of them consisting of twelve different temperatures of 50°C, 100°C, 150°C, 200°C, 250°C, 300°C, 350°C, 400°C, 450°C, 500°C, 550°C and 600°C for both SW and SBW. Average values were taken into consideration when observing the temperature effect. Three types of products have been obtained in these cases: liquid oil, solid char, and gaseous substances. Temperature effects on pyrolytic product yields of SW and SBW are shown in Figure 6, Figure 7**,** respectively.

Figure 6, Figure 7 show that as the temperature increases, the generation rate of the liquid product also increases until the temperature reaches around 450°C. However, for solid, the trend is the opposite. The increasing trend of oil production and decreasing char production trends have been seen for both raw materials. The trend is to some extent constant for gas production, but after reaching 450°C, the production of gas increases. Three sets of experiments were conducted, and an increasing trend has been found for liquid production, and maximum liquid yield was found at 450°C. The maximum yield (by weight) of pyrolytic oil from these experiments with SW was 53.2% at a temperature of 450°C, and the maximum yield (by weight) of pyrolytic char from SW was 73.1% at a temperature of 50°C. Similarly, the maximum yield (by weight) of pyrolytic oil from these experiments with SBW was 54.9% at a temperature of 450°C and the maximum yield (by weight) of pyrolytic char from SBW was 72.1% at a temperature of 50°C.

### 3.4 Characterization of pyrolytic oil by GC–MS analysis

**Figure 8:**
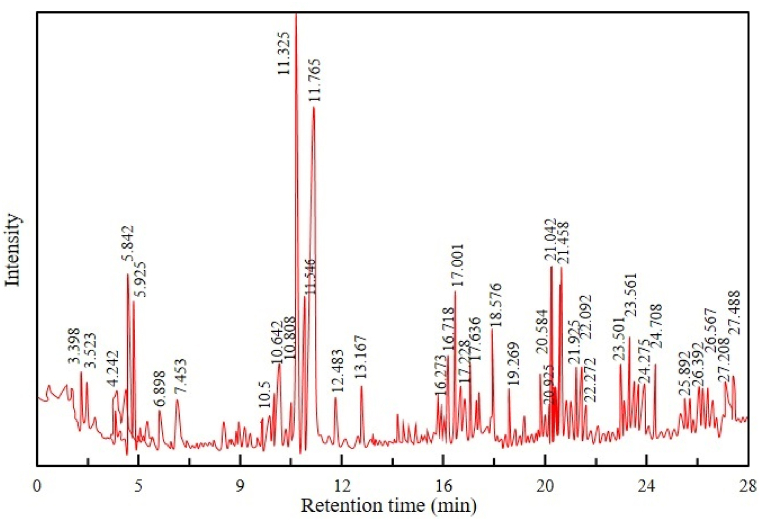
Chromatographic analysis of pyrolytic oil derived from SW

**Figure 9:**
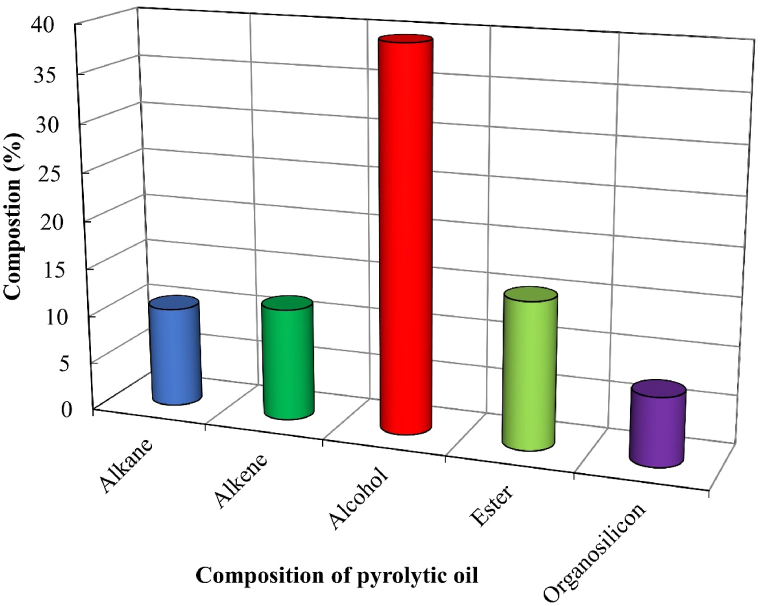
Compositional comparison of pyrolytic oil derived from SW

The major products of SW and SBW were condensable liquids. GC-MS analysis is a very resourceful process as it quantifies the components effectively. GC-MS analysis was carried out for pyrolytic oil obtained from this study. The purpose of the test was to get an idea about the characteristics and categories of the compounds of evolved pyrolytic oils and to find some promising methods of treating and recycling those oils [52]. The analysis was conducted at Dhaka's Bangladesh Council of Scientific and Industrial Research (BCSIR) laboratory. Fig. 8, Table 3, and Fig. 9 show, respectively, the chromatographic analysis, composition, and compositional comparison of pyrolytic oil derived at 450°C from SW.

**Table 3:** Composition of pyrolytic oil derived from SW

Types of Compounds	Name of Components	Chemical Formula	Boiling Point (^°^C)	GC-MS Data	
				Components (%, peak area)	Retention Time (min)
Alkane	dodecane, 4,6-dimethyl-	C_14_H_30_	235.5	1.06	10.642
	decane, 1-iodo	C_10_H_21_I	132	0.80	16.273
	eicosane	C_20_H_42_	343	0.94	17.383
	eicosane	C_20_H_42_	343	1.68	20.584
	eicosane	C_20_H_42_	343	1.68	20.584
	1-cyclopentyleicosane	C_25_H_50_	513	1.03	20.925
	1-cyclopentyleicosane	C_25_H_50_	513	1.08	21.675
	tetrapentacontane, 1,54-dibromo-	C_54_H_108_Br_2_	830	0.81	26.392
	tetrapentacontane	C_54_H_110_	596	1.65	23.501
	11-methyltricosane	C_24_H_50_		1.31	23.991
Alkene	2-decene, 4-methyl-, (z)-	C_11_H_22_	218	2.84	4.242
	2-undecene, 4,5-dimethyl-, [r∗,s∗-(z)]-	C_13_H_26_	–	4.33	5.925
	9-eicosene, (e)-	C_20_H_40_	–	2.44	10.808
	heptacos-1-ene	C_27_H_54_	415	1.00	21.175
	1-nonadecene	C_19_H_38_	181	1.02	21.781
Alcohol	n-tridecan-1-ol	C_13_H_28_O	155	0.86	6.9
	(2,4,6-trimethylcyclohexyl) methanol	C_10_H_20_O	–	0.77	7.45
	1-decanol, 2-hexyl-	C_16_H_34_O	304	3.27	21.458
	1-decanol, 2-hexyl-	C_16_H_34_O	304	2.95	17.001
	1-decanol, 2-hexyl-	C_16_H_34_O	304	1.11	17.228
	1-decanol, 2-hexyl-	C_16_H_34_O	304	1.84	17.636
	11-methyldodecanol	C_13_H_28_O	261	4.44	11.55
	11-methyldodecanol	C_13_H_28_O	261	8.72	11.767
	1-heptanol, 2,4-diethyl-	C_11_H_24_O	223	1.01	12.483
	1-decanol, 2-hexyl-	C_16_H_34_O	304	1.66	21.925
	10-dodecen-1-ol, 7,11-dimethyl-	C_14_H_28_O	–	1.77	22.092
Ester	butyric acid, 2-phenyl-, dec-2-yl ester	C_20_H_32_O_2_	–	1.61	10.5
	hexa-triacontyl-trifluoroacetate	C_38_H_73_F_3_O_2_	867	3.70	21.042
	nonadecylpentafluoropropionate	C_22_H_39_F_5_O_2_	496	0.81	21.567
	triacontylheptafluorobutyrate	C_34_H_61_F_7_O_2_	–	1.63	23.858
	octatriacontylpentafluoropropionate	C_41_H_77_F_5_O_2_	641	1.52	24.158
	ethyl 14-methyl-hexadecanoate	C_19_H_38_O_2_	–	1.02	24.275
Organosilicon	cyclononasiloxane, octadecamethyl-	C_18_H_54_O_9_Si_9_	416	1.93	27.488
	cyclodecasiloxane, eicosamethyl-	C_20_H_60_O_10_Si_10_	452	1.52	29.330
	cyclodecasiloxane, eicosamethyl-	C_20_H_60_O_10_Si_10_	452	1.40	31.194
	tetracosamethyl-cyclodecasiloxane	C_24_H_72_O_12_Si_12_	518	1.46	33.140
	cyclononasiloxane, octadecamethyl-	C_18_H_54_O_9_Si_9_	416	0.74	22.272
Cyclic hydrocarbon	cyclohexane, 1,2,3,5-tetraisopropyl-	C_18_H_36_	–	0.74	23.633
	cyclohexane, 1,2,3,5-tetraisopropyl-	C_18_H_36_	–	1.11	24.35
	cyclohexane, 1,2,3,5-tetraisopropyl-	C_18_H_36_	–	1.02	24.708
	cyclohexane, 1,2,3,5-tetraisopropyl-	C_18_H_36_	–	2.43	27.208
Ether	hexacosylnonyl ether	C_35_H_72_O	–	0.82	25.892
Thiol	tert-hexadecanethiol	C_16_H_34_S	329	1.19	26.567

It is seen from Table 3 that evolved pyrolytic oil has a higher concentration of some valuable compounds, namely 1-decanol, 2-hexyl-, which is used in producing surfactants, emulsifiers, plasticizers, lubricants, solvents and aroma additives, 11-Methyldodecanol which is used in perfume, flavor, cosmetics and surfactants industries and Hexa-triacontyl-trifluoroacetate which is used in preparing surface coatings, protective films and in research etc. Other researchers have found similar types of compounds [8,37,41,53,54]. Other identified compounds in this research are often used in various industrial applications.

Fig. 9 shows the compositional comparison, which indicates a higher presence of alcoholic compounds in pyrolytic oil derived from SW. Esters, alkenes, alkanes, and organosilicons are also present in the produced pyrolytic oil.

**Figure 10:**
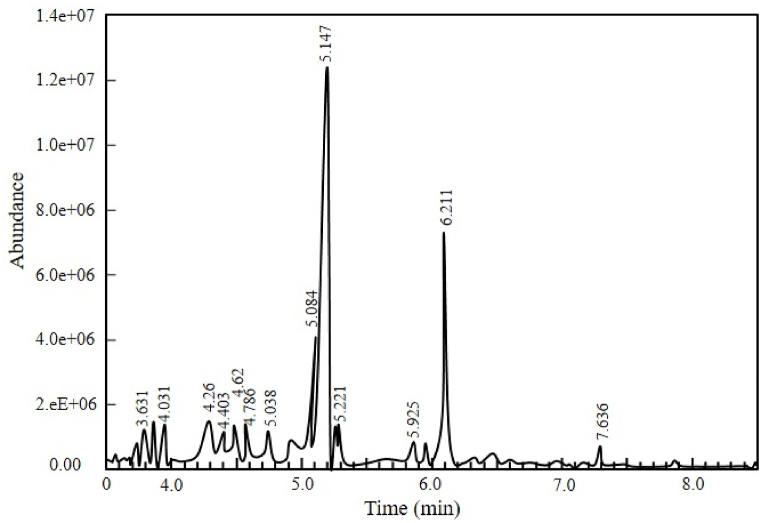
Chromatographic analysis of pyrolytic oil derived from SBW

**Figure 11:**
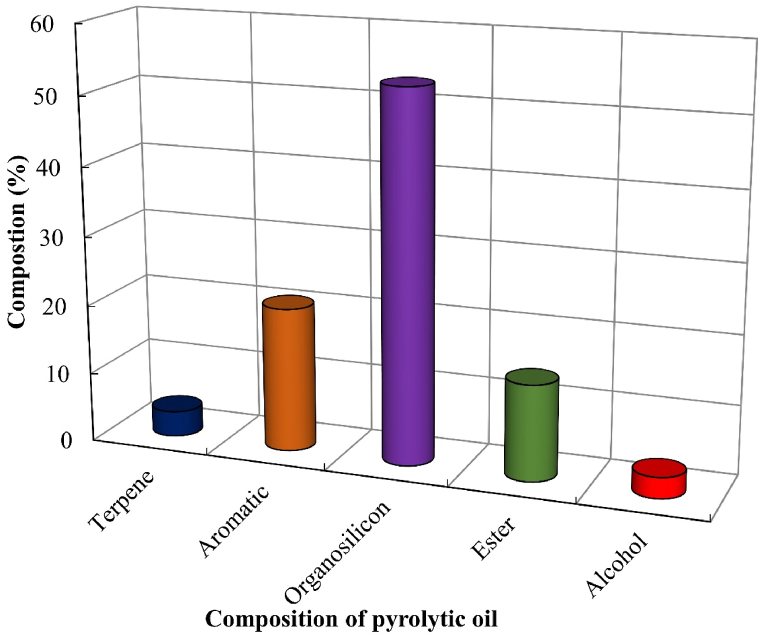
Compositional comparison of pyrolytic oil derived from SBW

Fig. 10, Table 4, and Fig. 11 show, respectively, the chromatographic analysis, composition, and compositional comparison of pyrolytic oil derived at 450°C from SBW.

**Table 4:** Composition of pyrolytic oil derived from SBW

Types of Compounds	Name of Components	Chemical Formula	Boiling Point (^°^C)	GC-MS Data	
				Components (%, peak area)	Retention Time (min)
Terpene	.alpha.-bisabolol oxide b	C_15_H_26_O_2_	326	3.66	3.419
Aromatic	2-methyl-5h-dibenz[b,f]azepine	C_19_H_17_N	359	1.68	3.631
	oxime-, methoxy-phenyl-	C_8_H_9_NO_2_	–	7.29	4.031
	benzene, 1,4-bis(trimethylsilyl)-	C_12_H_24_Si_2_	194	1.98	5.038
	6 methyl-2 phenylindole	C_15_H_15_N	397	8.21	5.084
	benzoic acid	C_7_H_6_O_2_	250	1.62	5.925
Organophosphorus	2-(dimethylamino)-1,3-dimethyltetrahydro-1,3,2-diazaphosphole 2-oxide	C_7_H_13_N_2_O_2_P	–	1.81	4.26
ketone	2-octanone	C_8_H_16_O	173	2.32	4.403
Alcohol	ethylhexanol	C_8_H_18_O	185	1.61	4.786
	2,5-hexanediol, 2,5-dimethyl-	C_8_H_16_O_2_	214	1.39	5.221
Organosilicon	1,1,3,3,5,5-hexamethyl-cyclohexasiloxane	C_6_H_18_O_6_Si_6_	–	10.64	3.51
	octamethylcyclotetrasiloxane	C_8_H_24_O_4_Si_4_	175	2.37	4.62
	3,3-diisopropoxy-1,1,1,5,5,5-hexamethyltrisiloxane	C_14_H_32_O_5_Si_3_	–	40.11	5.147
Ester	perhydro-htx-2-one, 2-depentyl-, acetate ester	C_13_H_24_O_3_	–	13.81	6.211
Ether	3,4-dihydroxybenzyl alcohol, tris(trimethylsilyl)-	C_17_H_32_O_3_Si_3_	363	1.48	7.636

It is seen from Table 4 that evolved pyrolytic oil has a higher concentration of hexamethyl-cyclohexasiloxane which is used in cosmetics, lubricants and producing silicone polymers, Oxime-, methoxy-phenyl- which is used in chemical synthesis and pharmaceuticals, 6 methyl-2 phenylindole which is used in pharmaceutical research and organic synthesis, 3,3-Diisopropoxy-1,1,1,5,5,5-hexamethyltrisiloxane which is used in preparing surface treatments and cosmetics, Perhydro-htx-2-one which is used in perfume industry and solvents, 2-depentyl-, acetate ester which is used in fragrance industry and solvents etc. Other researchers have found similar types of compounds [8,53,54]. Other detected compounds in this research are often used in various industrial applications.

Fig. 11 shows the compositional comparison, which suggests a higher presence of organosilicons in pyrolytic oil derived from SBW. Aromatics, esters, terpenes, and alcohols are also present in the produced pyrolytic oil.

### 3.5 Characterization of pyrolytic oil by FT-IR

**Figure 12:**
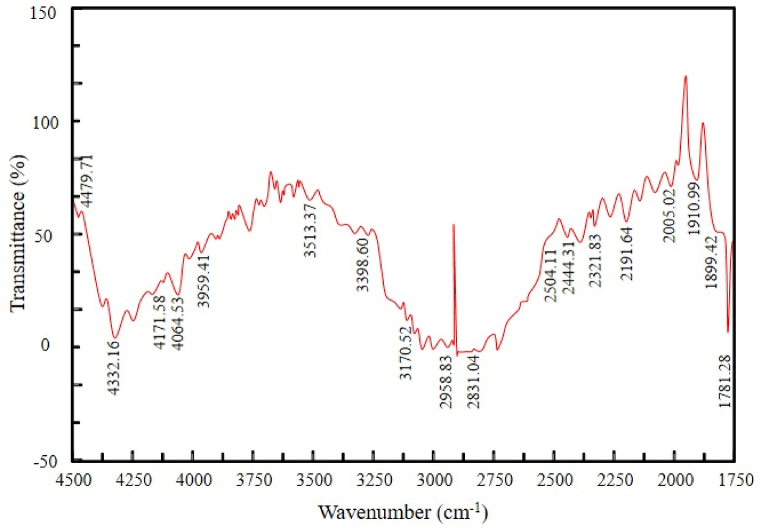
FT-IR spectra of pyrolytic oil derived from SW

**Figure 13:**
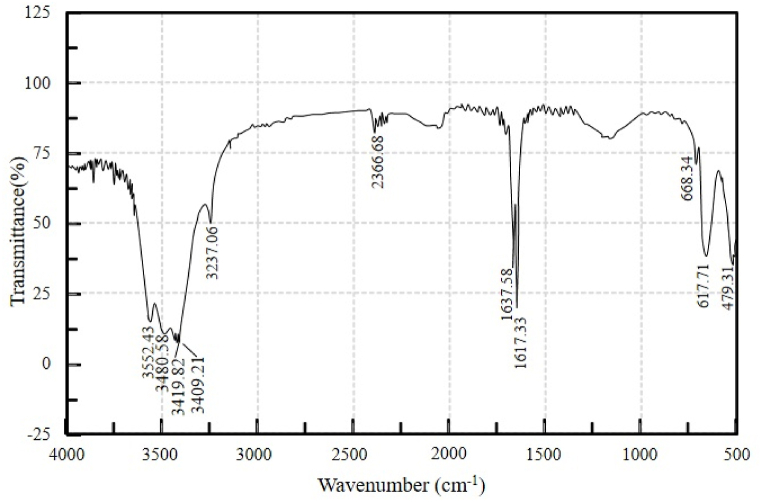
FT-IR spectra of pyrolytic oil derived from SBW

Fourier-transform infrared spectroscopy (FT-IR) provides qualitative and quantitative information about organic and inorganic samples. It determines the class of chemical compounds and associated functional groups. The analysis was conducted at Dhaka's Bangladesh Council of Scientific and Industrial Research (BCSIR) laboratory. Figure 12, Figure 13 show the FT-IR spectra of pyrolytic oil derived at 450°C from SW and SBW, respectively.

In Fig. 12, C=O stretching vibrations at frequency 1781.28 indicate the presence of carboxylic acids. The presence of alkynes is identified at 2191.64 cm^−1^ with ‒C ≡ C‒ stretching vibrations. Alkanes are present at 2958 cm^−1^ with C‒H stretching vibrations. The presence of alcohols is detected at 3393.78 cm^−1^ and 3398.6 cm^−1^ with O‒H starching and H-bonded vibrations. A similar functional group of compounds has been found in the oil generated from the pyrolysis of disposable syringes [37,41]. The results were well-matched when juxtaposed with the results of GC-MS and indicative to Polypropylene and Polyethylene polymer.

In Fig. 13, C‒Cl stretching vibrations at frequency 668.34 indicate the presence of alkyl halides. The presence of alkenes is identified at 1637.58 cm^−1^ with C=C stretching vibrations. The presence of alcohols and phenols is discovered at 3237.06 cm^−1^, 3409.21 cm^−1^, 3412.59 cm^−1^, 3419.82 cm^−1^, and 3480.58 cm^−1^ with O‒H stretching and H-bonded vibrations. The results were well-matched when juxtaposed with the results of GC-MS and declarative to Polypropylene and Polyethylene polymer. Table 5 summarizes the characteristic absorptions (cm^−1^) determined in the spectra.

**Table 5:** FT-IR functional groups and compounds of pyrolytic oil from SW and SBW

Pyrolytic Oil from SW (Characteristic Absorptions, cm^−1^)	Pyrolytic Oil from SBW (Characteristic Absorptions, cm^−1^)	Functional Group	Classification of Compounds
–	600–800	C-Cl	alkyl halides
–	1620–1680	C=C	alkenes
1670–1820	–	C=O (stretch)	carbonyls
2100–2260	–	‒C ≡ C‒ (stretch)	alkynes
2850–3000	–	C‒H (stretch)	alkanes
3200–3600	3200–3600	O‒H (stretch, H-bonded)	alcohols and phenols

### 3.6 Comparison of properties of evolved pyrolytic oil with other commercial fuels

The characteristic properties of the evolved pyrolytic oil from two different types of medical waste are essential for understanding the qualitative value of the pyrolytic liquid. As we got the maximum liquid yield at 450°C, the SW and SBW oil samples used for comparison were obtained from this reaction temperature. These properties are presented in a tabular form in Table 6.

**Table 6:** Property comparison between pyrolytic oils derived from SW, SBW, Gasoline and Diesel

Physical Properties	SW	SBW	Gasoline [41,54]	Diesel [41,54]
Density (kg/m^3^)	758	726	720	840
Kinematic Viscosity at 40 °C (cS)	4.75	3.19	0.6	2–5.5
Pour Point (^°^C)	−12	−16	−40	−40 to −1
Boiling Point (^°^C)	95	86	27 to 225	172 to 355
Cloud Point (^°^C)	−2	−5	–	−9
Gross Calorific Value (MJ/kg)	41.519	43.578	42 to 46	42 to 45

It is noted from Table 6 that the density of pyrolytic oil derived from SW and SBW is quite like gasoline but it is lesser than diesel. Kinematic viscosity is slightly higher than gasoline but comparable to diesel. The pour, boiling, and cloud points are identical to gasoline and diesel. The GCV of pyrolytic oil obtained from SW and SBW in this research is like the GCV of gasoline and diesel. After some pretreatment process pyrolytic oils from SW and SBW could be used in various industrial applications.

### 3.7 GCV of syringe waste and saline bottle waste in different states

GCV of SW and SBW in different states (i.e., raw waste, pyrolytic oil, and char) were measured. Table 7 summarizes the heating values of SW and SBW in various conditions.

**Table 7:** GCV of SW and SBW in different states

Types of Waste Materials	GCV of Raw Waste (MJ/kg)	GCV of Pyrolytic Oil (MJ//kg)	GCV of Pyrolytic Char (MJ/kg)
SW	41.06	41.519	42.737
SBW	43.31	43.578	43.319

Table 7 shows that the calorific value of evolved pyrolytic oil and char from SW is slightly higher than the calorific value of raw SW. The calorific value of evolved pyrolytic oil from SBW is slightly higher than the calorific value of raw SBW, but the GCV of raw SBW and char is comparable. GCV of raw SW is less than that of SBW, which is supported by the fixed carbon percentage shown in Table 2, but both values are similar to petrol or diesel oil [41].

## 4 Conclusion

A fixed bed reactor was designed and constructed for pyrolysis of SW and SBW to convert these wastes into solid, liquid, and gaseous products. The maximum production of pyrolytic oil and char from SW was 53.2% at 450°C and 73.1% at 50°C, respectively. Similarly, the maximum production of pyrolytic oil and char from SBW was 54.9% at 450°C and 72.1% at 50°C respectively. The process could reduce the difficulty of disposing of waste syringes and saline bottles as well as be a source of alternative energy and chemicals. Future research recommendations of measuring the effect of heating rate, feedstock size, residence time, and different types of reactors on product yield were strongly suggested. GC-MS analysis of pyrolytic oil from both SW and SBW shows the presence of beneficial chemical compounds. FT-IR tests supported the results of GC-MS and indicated a noticeable number of hydrocarbons in both SW and SBW pyrolytic oil. Various types of catalysts were highly recommended for upgrading the quality of pyrolytic products. Solid char obtained from SW and SBW could also be used as fuel after making them solid lumps by mixing with some binder or can be utilized as activated carbon. Pyrolysis of SW and SBW also decreases the environmental vulnerabilities resulting from the unmanaged disposal of such waste. Thus, proper management of plastic-based medical waste is also recommended through this research.

## CRediT authorship contribution statement

**Adnan Abedeen:** Writing – Original draft, review & editing, methodology, data analysis. **Md. Shameem Hossain:** Writing – review & editing, data analysis, methodology, conceptualization, Supervision. **A.N.M. Mizanur Rahman:** Writing – review and editing, methodology and Supervision.

## Data availability statement

All the data required are available within the manuscript.

## Declaration of competing interest

The authors declare that they have no known competing financial interests or personal relationships that could have appeared to influence the work reported in this paper.

## Nomenclature

BCSIR: Bangladesh Council of Scientific and Industrial Research
CARS: Centre for Advanced Research in Sciences
CHNS: Carbon, Hydrogen, Nitrogen and Sulfur
DTG: Differential Thermogravimetry
DU: Dhaka University
FT-IR: Fourier-Transform Infrared Spectroscopy
GC-MS: Gas Chromatography–Mass Spectrometry
GCV: Gross Calorific Value
HCW: Healthcare Waste
HCWGR: Healthcare Waste Generation Rate
HDPE: High-density Polyethylene
KCC: Khulna City Corporation
KUET: Khulna University of Engineering & Technology
PE: Polyethylene
PET: Polyethylene Terephthalate
PMW: Plastic Medical Waste
PP: Polypropylene
PS: Polystyrene
RCC: Rajshahi City Corporation
SW: Syringe Waste
SBW: Saline Bottle Waste
TGA: Thermogravimetric Analysis

## References

[1] Panda A.K., Singh R.K., Mishra D.K. (2010). Thermolysis of waste plastics to liquid fuel: a suitable method for plastic waste management and manufacture of value-added products-A world perspective. Journal of Renewable and Sustainable Energy Reviews.

[2] Cheng Y.W., Sung F.C., Yang Y., Lo Y.H., Chung Y.T., Li K.C. (2009). Medical waste production at hospitals and associated factors. Journal of Waste Management.

[3] Hassan M.F., Shareefdeen Z. (2022). Recent developments in sustainable management of healthcare waste and treatment technologies. Journal of Sustainable Development of Energy, Water and Environment Systems.

[4] Sartaj M., Arabgol R. (2015). Assessment of healthcare waste management practices and associated problems in Isfahan Province (Iran). J. Mater. Cycles Waste Manag..

[5] Diaz L.F., Eggerth L.L., Enkhtsetseg S.H., Savage G.M. (2008). Characteristics of healthcare wastes. Waste management.

[6] Komilis D.P. (2016). Issues on medical waste management research. Journal of Waste Management.

[7] Mbongwe B., Mmereki B.T., Magashula A. (2008). Healthcare waste management: current practices in selected healthcare facilities, Botswana. Journal of Waste Management.

[8] Som U., Rahman F., Hossain S. (2018). Recovery of pyrolytic oil from thermal pyrolysis of medical waste. Journal of Engineering Sciences.

[9] Lopez A., De Marco I., Caballero B.M., Laresgoiti M.F., Adrados A. (2011). Influence of time and temperature on pyrolysis of plastic wastes in a semi-batch reactor. J. Chem. Eng..

[10] Dai L., Zhou N., Lv Y., Cheng Y., Wang Y., Liu Y., Cobb K., Chen P., Lei H., Ruan R. (2022). Pyrolysis technology for plastic waste recycling: a state-of-the-art review. Prog. Energy Combust. Sci..

[11] Patwary M.A., O'Hare W.T., Street G., Elahi K.M., Hossain S.S., Sarker M.H. (2009). Quantitative assessment of medical waste generation in the capital city of Bangladesh. Journal of Waste Management.

[12] Basak S.R., Mita A.F., Ekra N.J., Alam M.J.B. (2019). A study on hospital waste management of Sylhet city in Bangladesh. International Journal of Engineering Applied Sciences and Technology.

[13] Patil A.D., Shekdar A.V. (2001). Healthcare waste management in India. J. Environ. Manag..

[14] Windfeld E.S., Brooks M.S.L. (2015). Medical waste management–A review. J. Environ. Manag..

[15] Rabeie O.L., Miranzadeh M.B., Fallah S.H., Dehqan S., Moulana Z., Amouei A., Mohammadi A.A., Asgharnia H.A., Babaie M. (2012). Determination of hospital waste composition and management in Amol city, Iran. Journal of Health Scope.

[16] Ananth A.P., Prashanthini V., Visvanathan C. (2010). Healthcare waste management in Asia. Journal of Waste Management.

[17] Tantanee S., Hantrakul S. (2019). Municipal waste management challenge of urbanization: lesson learned from phitsanulok, Thailand. Geogr. Tech..

[18] Bazrafshan E., Kord Mostafapoor F. (2011). Survey of medical waste characterization and management in Iran: a case study of Sistan and Baluchestan Province. Journal of Waste Management & Research.

[19] Phengxay S., Okumura J., Miyoshi M., Sakisaka K., Kuroiwa C., Phengxay M. (2005). Healthcare waste management in Lao PDR: a case study. Journal of Waste Management & Research.

[20] Yong Z., Gang X., Guanxing W., Tao Z., Dawei J. (2009). Medical waste management in China: a case study of Nanjing. Journal of Waste management.

[21] Gai R., Kuroiwa C., Xu L., Wang X., Zhang Y., Li H., Zhou C., He J., Tang W., Kuroiwa C., Tang W. (2009). Hospital medical waste management in Shandong Province, China. Journal of Waste management & research.

[22] Hossain M.S., Santhanam A., Norulaini N.N., Omar A.M. (2011). Clinical solid waste management practices and its impact on human health and environment-A review. Journal of Waste Management.

[23] Eker H.H., Bilgili M.S. (2011). Statistical analysis of waste generation in healthcare services: a case study. Journal of Waste Management & Research.

[24] Budati S., Reddy A.P., Prasad N.R., Shaik N. (2024). Management of COVID-19 medical waste based on pyrolysis-swot analysis. Int J Acad Med Pharm.

[25] Kantová N.Č., Cibula R., Szlek A., Čaja A., Nosek R., Belany P. (2023). The energy assessment of COVID-19 medical waste as a potential fuel. Energy Rep..

[26] Choudhary R., Mukhija A., Sharma S., Choudhary R., Chand A., Dewangan A.K., Gaurav G.K., Klemeš J.J. (2023). Energy-saving COVID–19 biomedical plastic waste treatment using the thermal-Catalytic pyrolysis. Energy.

[27] Ramalingam S., Thamizhvel R., Sudagar S., Silambarasan R. (2023). Production of third generation bio-fuel through thermal cracking process by utilizing Covid-19 plastic wastes. Mater. Today Proc..

[28] Dharmaraj S., Ashokkumar V., Pandiyan R., Munawaroh H.S.H., Chew K.W., Chen W.H., Ngamcharussrivichai C. (2021). Pyrolysis: an effective technique for degradation of COVID-19 medical wastes. Chemosphere.

[29] Chen C., Chen J., Fang R., Ye F., Yang Z., Wang Z., Shi F., Tan W. (2021). What medical waste management system may cope with COVID-19 pandemic: lessons from Wuhan. Resour. Conserv. Recycl..

[30] Su G., Ong H.C., Ibrahim S., Fattah I.R., Mofijur M., Chong C.T. (2021). Valorisation of medical waste through pyrolysis for a cleaner environment: progress and challenges. Environmental Pollution.

[31] Jeba J.S., Rahman M.M. (2022). Medical waste management in Khulna city corporation, Bangladesh. Natl. Geogr. J. India.

[32] Rahman M.S., Moumita C., Rikta K. (2013). Medical waste management system: an alarming threat (A case study on jessore municipality, Bangladesh). Journal of Environmental Science and Natural Resources.

[33] Alam M.Z., Islam M.S., Islam M.R. (2013). Medical waste management: a case study on Rajshahi city corporation in Bangladesh. Journal of Environmental Science and Natural Resources.

[34] Ullah F., Ji G., Zhang L., Irfan M., Fu Z., Manzoor Z., Li A. (2023). Assessing pyrolysis performance and product evolution of various medical wastes based on model-free and TG-FTIR-MS methods. Chem. Eng. J..

[35] Xu W., Liu J., Ding Z., Fu J., Evrendilek F., Xie W., He Y. (2022). Dynamic pyrolytic reaction mechanisms, pathways, and products of medical masks and infusion tubes. Sci. Total Environ..

[36] Ding Z., Chen H., Liu J., Cai H., Evrendilek F., Buyukada M. (2021). Pyrolysis dynamics of two medical plastic wastes: drivers, behaviors, evolved gases, reaction mechanisms, and pathways. J. Hazard Mater..

[37] Vasile C., Brebu M., Darie H., Deanin R.D., Dorneanu V., Pantea D.M., Ciochina O.G. (1997). Chemicals and energy from medical polymer wastes I. Pyrolysis of disposable syringes. Int. J. Polym. Mater..

[38] Deng N., Zhang Y.F., Wang Y. (2008). Thermogravimetric analysis and kinetic study on pyrolysis of representative medical waste composition. Journal of Waste Management.

[39] Zhu H.M., Yan J.H., Jiang X.G., Lai Y.E., Cen K.F. (2008). Study on pyrolysis of typical medical waste materials by using TG-FT-IR analysis. J. Hazard Mater..

[40] Bernardo M., Lapa N., Gonçalves M., Mendes B., Pinto F., Fonseca I., Lopes H. (2012). Physico-chemical properties of chars obtained in the co-pyrolysis of waste mixtures. J. Hazard Mater..

[41] Dash A., Kumar S., Singh R.K. (2015). Thermolysis of medical waste (Waste Syringe) to liquid fuel using semi-batch reactor. Journal of Waste and Biomass Valorization.

[42] Ahmad I., Khan M.I., Khan H., Ishaq M., Tariq R., Gul K., Ahmad W. (2015). Pyrolysis study of polypropylene and polyethylene into premium oil products. Int. J. Green Energy.

[43] Pramanik H., Gaurh P. (2019). Production and characterization of pyrolysis oil using waste polyethylene in a semi-batch reactor. Indian J. Chem. Technol..

[44] Williams P.T., Besler S. (1996). The influence of temperature and heating rate on the slow pyrolysis of biomass. Journal of Renewable energy.

[45] Jung S.H., Cho M.H., Kang B.S., Kim J.S. (2010). Pyrolysis of a fraction of waste polypropylene and polyethylene for the recovery of BTX aromatics using a fluidized bed reactor. Journal of Fuel Processing Technology.

[46] Khan M.Z.H., Sultana M., Al-Mamun M.R.A., Hasan M.R. (2016). Pyrolytic waste plastic oil and its diesel blend: fuel characterization. Journal of Environmental and Public Health.

[47] López A., de Marco I., Caballero B.M., Laresgoiti M.F., Adrados A. (2010). Pyrolysis of municipal plastic wastes: influence of raw material composition. Journal of Waste Management.

[48] Adrados A., De Marco I., Caballero B.M., López A., Laresgoiti M.F., Torres A. (2012). Pyrolysis of plastic packaging waste: a comparison of plastic residuals from material recovery facilities with simulated plastic waste. Journal of Waste Management.

[49] Brems A., Baeyens J., Vandecasteele C., Dewil R. (2011). Polymeric cracking of waste polyethylene terephthalate to chemicals and energy. J. Air Waste Manag. Assoc..

[50] Widiyannita A.M., Cahyono R.B., Budiman A., Sutijan, Akiyama T. (2016, July).

[51] Singh R.K., Ruj B., Sadhukhan A.K., Gupta P. (2019). Thermal degradation of waste plastics under non-sweeping atmosphere: Part 1: effect of temperature, product optimization, and degradation mechanism. J. Environ. Manag..

[52] Hossain M.S., Abedeen A., Karim M.R., Moniruzzaman M., Hosen M.J. (2017). Catalytic pyrolysis of waste tires: the influence of ZSM-catalyst/tire ratio on product. Iran. J. Energy Environ..

[53] Fang S., Jiang L., Li P., Bai J., Chang C. (2020). Study on pyrolysis product characteristics of medical waste and fractional condensation of the pyrolysis oil. Energy.

[54] Ullah F., Zhang L., Ji G., Irfan M., Ma D., Li A. (2022). Experimental analysis on product distribution and characterization of medical waste pyrolysis with a focus on liquid yield quantity and quality. Sci. Total Environ..

